# bims: Biomed News

**DOI:** 10.5195/jmla.2026.2288

**Published:** 2026-01-01

**Authors:** Farhad Shokraneh

**Affiliations:** 1 farhad.shokraneh@nottingham.ac.uk, Evidence Synthesis Manager, the University of Oxford; Research Fellow, the University of Bristol; and Post-Doc Research Associate, Cambridge University, UK

## Abstract

**bims: Biomed News**. February 5, 2017–Present. https://biomed.news/, Created by Thomas Krichel and directed by Gavin P. McStay. Free. Accessible via any web browser.

## DESCRIPTION

bims: Biomed News is a free weekly newsletter report creation system based on PubMed. The newsletters contain selected recent PubMed records that pertain to the report's topic. Therefore, bims can be categorized among the tools that provide Selective Dissemination of Information (SDI).

## USERS

bims community has two types of users: editors and readers. No criteria need to be met to become a reader or an editor.

**Editors** select the papers for the weekly report issues. Most editors appear to be biomedical researchers or systematic reviewers. Some are patients, staff, or patient support organizations. This is typically the audience that health librarians serve. Therefore, a review of this tool seems pertinent.

**Readers** can subscribe to weekly issues of any report. The reports are listed at https://biomed.news/reports. Each report has a “Sign Up” button. When you sign up, you get the weekly issues of the report in your inbox.

## COST

bims is an open and free tool with no monetary or non-monetary barrier to becoming an editor, nor to becoming a reader.

## HOW TO CREATE AND MAINTAIN A BIMS NEWSLETTER REPORT

**Opening a new newsletter report**. To become an editor, you need to open a report. This is done at https://biomed.news/open_a_report. This opens a simple form. It requires you to enter a title for the report, your name, your email address, and at least one PubMed paper that you think is relevant. Upon submission, the form data is sent to bims management. They create the report and assign a six-character identifier. They then send you credentials. For example, the author launched bims-arines (ARtificial INtelligence in Evidence Synthesis). Its first issue was released on September 29, 2024.

**Figure 1:**
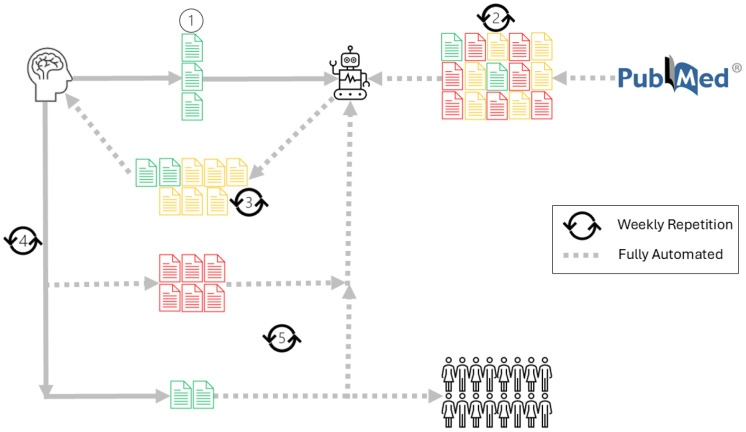
bims: Biomed News editors’ workflow. 1. Editor gives PMIDs of relevant records to bims. 2. bims automatically receives weekly records from PubMed. 3. bims automatically selects the possibly relevant records, sorts them based on relevancy, and sends them to the editor. 4. The editor selects the relevant ones; the rest of the records are automatically considered irrelevant and fed back to bims to improve its performance. 5. The relevant records are automatically sent to the editor, the readers, and bims to improve its performance.

**Maintaining the newsletter report**. When the report is opened, a weekly process starts. On Sunday at midnight UTC, you get an email alert that a new report issue, containing a list of possibly relevant new papers, is available for you to choose from. The new papers in PubMed are sorted by the likelihood that they are relevant to your topic. You select the relevant ones. The ones that you don't select are considered irrelevant. This data is fed into a bespoke AI tool. Thus, each week's data is used to improve the new selections. Over time, this leads to a selection of papers that is both more precise and more flexible than the ones found by searching tools (**Figure 1**).

**Output**. The relevant records selected by the editor are sent to the subscribed readers in the form of an email newsletter. The emails contain no advertisements. Each report issue is also published on a dedicated web page. At the time of writing, the author has published 44 weekly issues of bims-arines, reaching 83 subscribers. Every week, the author spends five minutes screening the received records, selecting them, and submitting them, ensuring subscribers stay up to date on the topic. The latest issues are found at http://biomed.news/bims-arines/latest.

## HOW TO USE THE EXISTING BIMS NEWSLETTER REPORTS

Readers use the site: https://biomed.news/.

**Cookies**. The site does not request permission to use cookies. Presumably, the site does not use cookies. It respects users’ privacy. This is an important issue for librarians when recommending a tool.

**User interface**. The site does not use gratuitous graphics or meaningless marketese. Instead, the homepage tries to convey the site's purpose through simple factual statements.

**Newsletter reports**. From the homepage, the most important link is the one to the reports, https://biomed.news/reports. The reports page lists all the active reports. Inactive reports can still be accessed. Next to each report, there is the title of the report. The title is an anchor to the latest issue of the report. Then, there is the editor's name. Usually, the name serves as the link to the editor's homepage. After the name, for most editors, the editor's affiliation appears, always with a link to the homepage of the organization, giving credit to both the editors and their organization.

**Sign Up**. Readers can sign up for the reports on https://biomed.news/reports or each report's page (bims-arines). The editors cannot see the list of subscribers, which is another positive privacy point.

**Unsubscribe**. Another user-friendly feature is that any report issue that readers receive includes an unsubscribe link, both in the email header and at the end of the email body, making it effortless to unsubscribe.

## COMMUNITY BUILDING AND ENGAGEMENT

**Timesaving through expertise-sharing**. An expert can create a newsletter on a specific topic of interest, and community members can subscribe to it, receiving updates every week. This way, rather than all community members searching for papers on the topic, only one expert member spends little time selecting the papers, and all community members benefit. Thus, bims is an expertise-sharing system.

**Crediting the editors**. Many social media platforms prevent or discourage linking one's profile or affiliated organization on the platform because such links direct readers away from the platform. Social media platforms all try to keep users on the platform. bims management credits the editors by mentioning their names and allowing them to link to their personal profiles and affiliated organizations.

**Free and open, shareable, and reusable reports**. The readers can use the report issue data in any way they see fit. For example, the weekly issue of the Biomed News report bims-librar on “Biomedical librarianship” is distributed to the MEDLIB-L mailing list, a community of over 1000 members.

## COMPARISON TO OTHER SDI SERVICES

While libraries and users have been utilizing binary SDI tools, such as email alerts for saved searches, new articles, or journals’ table of contents, bims offers a unique platform for leveraging machine learning to capture non-binary and fuzzy relevant papers that would otherwise be missed in a search. The author has summarized the key differences in **Table 1**.

**Table 1:** Comparing Selective Dissemination of Information (SDI) Methods

Features \ Method	Email Alerts or RSS			bims
	New Article	Table of Contents	Saved Boolean Search	
Frequency	ASAP	ASAP	Varies	Weekly
Specificity	✘	✘	✘/✔	✔
Output selectivity	✘	✘	✘/✔	✔
Output relevancy	✘/✔	✘/✔	✘/✔	✔
Machine learning	✘	✘	✘	✔
Human-in-the-loop	✘	✘	✘	✔
Community service	✘	✘	✘	✔

## ADVANTAGES

**Unprecedented performance**. bims has incomparably better performance compared to saved Boolean search email alerts. The author has set a saved Boolean search alert as well as a bims report on the same topic. bims outperforms in finding unique, new, relevant records.

**Users first**. Not using cookies or advertisements, offering an easy ‘unsubscribe’ option, and not sharing readers’ email addresses, even with the editors, demonstrate respect for users’ privacy and preferences.

**Expertise-saving**. In any other SDI methods, the expertise put to sort records as relevant and irrelevant at each email alert or RSS feed goes to waste. bims collects feedback from experts’ choices to improve its performance and save the editor's time in the next rankings.

**Ease of use**. Creating and maintaining the report, and subscribing and unsubscribing, are easy. All technical and complex aspects are left behind the scenes. Shortcut keys enable rapid movement across records for the editor without requiring the use of the mouse.

**User support**. bims management is directly and rapidly available via email. There are no chatbots or layers of bureaucracy.

**Potential use cases**. While the focus of this review was on using bims to keep the community up to date, it can also have other use cases, such as focusing on machine learning, including systematic review updates.

## LIMITATIONS

**Search facility**. bims lacks a search feature to locate and retrieve records on its website.

**Source of data**. Currently, PubMed is the only source of records for bims; however, the name ‘Biomed News’ suggests its possible generalizability to other accessible sources.

**Dependency on editors**. bims is not a regimented service. Editors can produce report issues at their leisure. Some may be very punctual, and others less so. Thus, subject report issues may appear at irregular intervals. Although it has been functioning well since 2017, community editors are crucial to the creation and maintenance of the reports. Reports can become inactive if the volunteer editor decides not to select the relevant records every week.

**Long-term interest**. Creating newsletter reports is suitable only for topics with long-term interest, where the editor is willing to invest a few minutes of their time per week in the topic.

**Subject coverage**. Uptake of bims varies across subject areas of PubMed. Some readers may not find a report on their topic of interest to sign up for.

**Quality control**. There is no quality control over the editors. A report can be created by anyone, including those who are completely unfamiliar with the topic. However, since maintenance of a report requires weekly action, the author presumes that inept editors would drop out quite soon.

**Specificity**. bims, as an SDI tool, may be unsuitable for broad topics (e.g., stroke, cancer); however, it works best with more specific topics and serves as a complementary tool to the existing Current Awareness Services (CAS) in libraries.

**Lack of email confirmation**. Upon signing up, users do not receive a confirmation email to verify their address. Thus, if you enter an invalid address, you will not see any report issues.

## CONCLUSION

bims is one of the rare modern and AI-based tools that advocates all Ranganathan's five laws of library science, making records usable, referring right records to the right readers, saving readers' and editors’ time, and saving the expert knowledge through feedback to the bims machine learning feature, improving its performance as an evolving and growing organism.

With knowledge of its use, advantages, and limitations, library and information science professionals can introduce bims to expert potential editors and readers with no concerns about the commercial use of their data or privacy issues.

